# How to Make Evolution-Proof Insecticides for Malaria Control

**DOI:** 10.1371/journal.pbio.1000058

**Published:** 2009-04-07

**Authors:** Andrew F Read, Penelope A Lynch, Matthew B Thomas

**Affiliations:** University of Bath, United Kingdom

## Abstract

The evolution of resistance to insecticides by mosquitoes is a major threat to ongoing malaria control programs and plans for global eradication. Evolutionary theory suggests a practical solution.

**Summary**

Insecticides are one of the cheapest, most effective, and best proven methods of controlling malaria, but mosquitoes can rapidly evolve resistance. Such evolution, first seen in the 1950s in areas of widespread DDT use, is a major challenge because attempts to comprehensively control and even eliminate malaria rely heavily on indoor house spraying and insecticide-treated bed nets. Current strategies for dealing with resistance evolution are expensive and open ended, and their sustainability has yet to be demonstrated. Here we show that if insecticides targeted old mosquitoes, and ideally old malaria-infected mosquitoes, they could provide effective malaria control while only weakly selecting for resistance. This alone would greatly enhance the useful life span of an insecticide. However, such weak selection for resistance can easily be overwhelmed if resistance is associated with fitness costs. In that case, late-life–acting insecticides would never be undermined by mosquito evolution. We discuss a number of practical ways to achieve this, including different use of existing chemical insecticides, biopesticides, and novel chemistry. Done right, a one-off investment in a single insecticide would solve the problem of mosquito resistance forever.

Indoor residual spraying (IRS) with insecticides continues to be a mainstay of malaria control, having been responsible for often spectacular reductions in disease incidence during the 20th century, including elimination of malaria from many countries [1–4]. More recently, insecticide-treated bed nets (ITNs) have become a leading tool for malaria control [4,5]. Major international efforts are currently underway to comprehensively control and even globally eradicate malaria, and these involve enormous up-scaling of IRS and ITN use [6–10]. As in the last century, one of the major challenges to these new efforts is the evolution of insecticide resistance in Anopheles populations [1,2,11–18]. IRS spraying for malaria was responsible for resistance evolution in countries as diverse as Greece, Java, Haiti, and Sudan [17,19–21]. Insecticide-resistant mosquitoes were one of the main hurdles faced by the ultimately unsuccessful Global Malaria Eradication plan in the middle of last century [1,2,11,13,14,17,22]. Contemporary experience is that nothing has changed. For instance, a surge in malaria cases from 600/month to 2,000/month in KwaZulu-Natal, South Africa, at the end of last century was associated with pyrethroid-resistant An. funestus [23,24]. In a recent 24-village trial in Mexico, the frequency of pyrethoid-resistant Anopheles went from effectively zero to 20% after three years of IRS (Box 1) [25]. There are also serious concerns [16–18,26–31] and increasing evidence [32–34] that insecticides on bed nets will similarly drive resistance evolution.

Once a “resistance crisis” [26] occurs, where disease control fails because mosquito evolution has rendered an insecticide ineffective, options are few, not least because of the very limited insecticide arsenal available. Insecticides recommended for malaria control by the World Health Organization (WHO) represent just four classes of compound for IRS and just one class of compounds for ITNs [13,15]. Consequently, there is an increasing focus on resistance management strategies, whereby efforts are made to use existing insecticides in ways which can maximize the time period for which they provide useful disease control (what we hereafter refer to as the “useful lifespan” of a compound). Resistance management strategies include the use of diverse insecticides in space and time (rotations and mosaics), insecticide mixtures, and restricting use to specific risk periods and locations [13,25,26,31,35–38]. Resistance management requires on-going surveillance [14,17] and a level of application management that is frequently problematic in regions where the malaria challenge is most severe. Moreover, techniques such as rotations and mixtures can be undermined by issues of cross resistance [13]. Indeed, given current restrictions on approved chemicals, there are virtually no options for resistance management for ITNs.

Consequently, there is now a concerted effort to identify new insecticidal compounds for use in malaria control [36,39]. On the face of it, this is desirable, but novel chemistry does not, in itself, provide a sustainable answer. All existing insecticides were “new” at some point, and there is the very real danger that, as with the antimalarial drug treadmill [40], the search for products can become open ended as the efficacy of successful new compounds is, in turn, eroded by the evolution of resistance. Here we show that the natural history of the Anopheles–Plasmodium interaction makes possible an alternative strategy to deal with insecticide resistance: the development of insecticides with properties that retard and even entirely prevent the spread of resistance. An “evolution-proof” compound would provide sustainable control, render conventional resistance management strategies unnecessary, and completely avoid an insecticide treadmill.

## The Proposition

All current insecticides approved for ITNs or IRS kill extremely rapidly after contact, and some are also irritants that cause the mosquito to move away from the net or house and search for blood meals elsewhere. Where coverage is high (a requirement for effective control), insecticides greatly reduce malaria transmission, but their high lethality or interference with blood feeding also imposes intense selection for resistance. It is our contention that effective transmission reduction can be achieved while minimizing selection for resistance. To simplify the following discussion, we initially consider only the lethal effects of insecticides; we return to the irritant (excito-repellency) effects at the end.

Our argument derives from the following observations. First, female mosquitoes convert a blood meal into eggs and oviposit in appropriate water bodies before seeking the next blood meal. This gonotrophic cycle takes 2–4 d [41,42]. Females contact insecticides on bed nets during feeding attempts, or on house walls while resting immediately after the feed. Second, extrinsic mortality rates for the key vector species, even in the absence of any public health measures, are very high—on the order of 10% per day or 20–40% per gonotrophic cycle [41,42]. The consequence is that most females go through only a few gonotrophic cycles before they die. Third, after infecting mosquitoes, malaria parasites go through various developmental stages and very many replicative cycles before migrating to the salivary glands, from where they can be transmitted to humans. The duration of this process (the sporogonic or extrinsic incubation period) depends on host, parasite, and environmental factors, but it is in the order of 10–14 d or 2–6 gonotrophic cycles in areas of high malaria transmission [41,42]. These facts together lead to one of the great ironies of malaria: most mosquitoes do not live long enough to transmit the disease.

These facts also mean that the majority of eggs a female will produce in her lifetime are laid in the window before malaria-infected mosquitoes can become dangerous to humans. Thus, in principle at least, public health advances can be achieved with minimal selection for resistance by an insecticide that kills after the majority of mosquito reproduction has occurred but before malaria parasites are infectious. Unlike in agriculture, the aim here is disease control, not necessarily insect control.

Below we consider how insecticides could be designed so as to kill only older mosquitoes, but we first compare the transmission control potential and the evolutionary properties of our proposed late-life–acting (LLA) insecticides with compounds like dichloro-diphenyl-trichloroethane (DDT), pyrethroids, and others currently in use (“conventional”' insecticides). The first key question is whether LLA insecticides can offer significant reductions in malaria transmission.

## Control

To assess the malaria control potential of LLA insecticides, we followed others [42–44] in developing a simple feeding cycle model (FCM) that deterministically tracks discrete cohorts of mosquitoes through their gonotrophic cycles, where mosquitoes have fixed probabilities of becoming infected with malaria parasites and, in our case, exposed to insecticides. The background mosquito mortality rates and durations of sporogony used to parameterize the baseline model are the average of four Plasmodium falciparum–endemic sites, two in Nigeria, one in Tanzania, and one in Papua New Guinea [42]. These sites are intense foci of malaria transmission.

An LLA insecticide could disproportionately kill older mosquitoes in two ways. First, it might work some time after first exposure (a time-dependent killer), as might be the case for an infectious agent. Second, the insecticide might be disproportionately effective against older mosquitoes, irrespective of time since contact (age-dependent killer), as might be the case if older insects are more physiologically vulnerable. In the following analysis, we consider this latter type of LLA insecticide, but our conclusions are unaltered in either case (Table S1).

The evolution of insecticide resistance is a practical problem only where insecticide coverage is high, which we take here to be 80%, a minimum target for coverage with IRS or ITNs [10]. For computational simplicity, we also assume that LLA insecticides have no impact on either total mosquito densities or the proportion of humans that are infectious. With these assumptions (and others, see Materials and Methods), we calculate that LLA insecticides killing mosquitoes that have reached 2 or more gonotrophic cycles will reduce the number of infectious bites by 99.2%. The corresponding figures for 3- and 4-cycle killers are 97.9% and 94.2%, respectively. These figures are highly encouraging, especially as they are minimum estimates: reductions in the number of infectious human cases following intervention will further reduce the number of infectious mosquitoes, as would higher or more-effective insecticide coverage and any effects on mosquito densities (more likely the earlier-acting the insecticide).

## Evolution

While fast-acting conventional insecticides can produce even more effective initial control (in our analysis, a 99.8% reduction in the number of infectious bites), they impose enormous selection for resistance by killing young female adults. The consequence is that spectacular initial mosquito control can last as little as a few years, thus providing very poor medium- to long-term disease control, as history has shown [22]. To analyze the evolutionary sustainability of LLA insecticides, we used fecundities calculated in our feeding cycle model as input into a discrete-time analog of standard population genetics models to track the spread of single-allele resistance through the population. Frequency of resistance in a population was calculated by assuming that resistance is dominant and ablates the mortality effects of the insecticide in question. We discuss the effect of relaxing the dominance assumption, and other assumptions, in Text S1.

**Figure 1:**
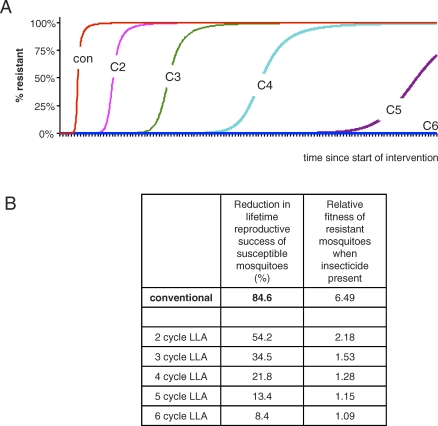
Evolutionary Consequences of Insecticides That Are Highly Lethal Immediately after First Contact (Conventional Insecticides, like DDT and Pyrethroids, Con) and Hypothetical LLA Insecticides That Kill Mosquitoes from Their Second through Sixth Gonotrophic Cycles (C2-C6) (A) Frequency of resistant mosquitoes through time. (B) Impact of insecticides on fitness of susceptible mosquitoes, and relative fitness of resistant mosquitoes in presence of insecticides, assuming no costs of resistance. LLA insecticides are a substantially less potent driver of the evolution of resistance than are conventional insecticides because of their susbstantially smaller impact on mosquito fitness. Note that when first deployed, four-cycle LLAs reduce the number of infectious bites by 94.2%. Two- and three-cycle killers remove more, but at cost of increased selection for resistance. We assume the control offered by five- and six-cycle killers, 76.6% and 57.1% of infectious bites removed, is too low to make them practicable (although absolute levels of control required will depend on local epidemiological context and the availability of other disease management tools). For model details and parameter values, see Materials and Methods.

With parameters as above, resistance spreads considerably more slowly for LLA insecticides than for conventional insecticides (Figure 1). This is because insecticides that kill on first contact will reduce mosquito lifetime reproductive success by about 85%. In contrast, insecticides that kill mosquitoes that have reached at least their fourth gonotrophic cycle eliminate just 22% of progeny (Figure 1). Thus, all else being equal, the fitness of a mutant resistant to conventional insecticides is 6.5 times that of the susceptible wild type; the corresponding advantage for a four-cycle killer is just 1.28.

The evolution of resistance to LLA insecticides could be slowed even further if they were disproportionately effective against malaria-infected mosquitoes. This is because insecticides that are less likely to kill uninfected mosquitoes further relax selection for resistance without any loss of control. For instance, if we leave the probability that a four-cycle LLA insecticide would kill infectious mosquitoes unaltered but halve its likelihood of killing uninfected mosquitoes, the time taken for resistance to reach 50% frequency would increase by about half as much again. A potentially useful side effect of disproportionate killing of malaria-infected mosquitoes would be to increase the selection pressure favoring malaria-resistant mosquitoes [45,46].

Importantly, resistance to LLA insecticides will not spread at all if there are nontrivial fitness costs to insecticide resistance. Reduced fitness of resistant insects in the absence of insecticides is frequently reported [47–49]. For Anopheles, costs of resistance have been seen in the laboratory [50,51] and, in the field, unexpectedly low frequencies of resistant homozygotes (e.g., [52]), and declines in resistance after withdrawal of causal insecticide (e.g., [18,25]) (see Box 1) point to substantial fitness costs. Costs of resistance have little impact on the evolution of resistance to conventional insecticides where the benefits of resistance are so high. The situation is, however, very different for LLA insecticides, where the fitness benefits of resistance (Figure 1) are very much lower. For LLA insecticides, resistance costs can outweigh resistance benefits, preventing resistance spreading at all, even when resistance alleles are present.

This argument follows from the evolutionary theory of aging [53–57]. The strength of selection declines with age. Beneficial genes that act late in life can fail to spread if they are associated with fitness costs earlier in life. This is because all individuals pay these costs, whereas only those few that survive to old age benefit. The theory of aging is well verified, not least in insects [58]. Senescence does occur in mosquito populations, and in Anopheles is detectable around the age at which mosquitoes can first become infectious to humans [59–62]. Thus, natural selection has not been strong enough to favor beneficial alleles that would act around the same time as would a putative resistance allele against a late-life insecticide.

**Figure 2:**
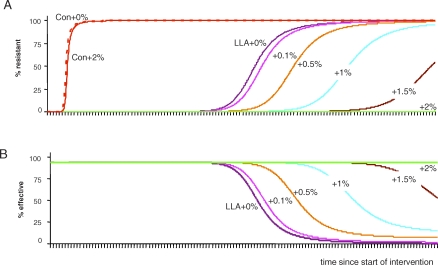
Evolutionary Consequences and Control Effectiveness of Insecticide Where There Are No Costs of Resistance (+0%) or Where the Costs of Resistance Accrue as Additional Daily Mortality Rates as Shown (A) Frequency of resistant mosquitoes through time. (B) Control effectiveness through time, where 0% effectiveness is the situation prior to the insecticide application, and 100% is complete absence of infectious bites. Plotted values are for a conventional insecticide (Con) or an LLA insecticide that kills mosquitoes on contact during or after their fourth gonotrophic cycle (a 4C LLA in Figure 1). Beyond the duration of our simulations, the LLA insecticide eventually fails even for a 2% cost of resistance (green line); for the parameter values used here, complete evolution-proofing occurs at 3.4%. For model details and parameter values, see Materials and Methods.

The inclusion of even modest costs of resistance substantially slows the rate at which resistance to LLA insecticides spreads in a population, thus considerably prolonging the effectiveness of malaria control (Figure 2). Importantly, it is also possible to maintain the initial levels of control forever. For the particular parameter values used here, costs of resistance, which accrue as an additional daily mortality rate of 3.4%, would render a four-cycle LLA insecticide completely evolution proof: this is the point at which the fitness gains of resistance, which benefit only a few, are outweighed by the fitness costs of resistance, which are paid by all. Thus, in principle at least, it is possible to create an insecticide that would provide effective malaria control yet never be undermined by the evolution of resistant mosquitoes.

**Figure 3:**
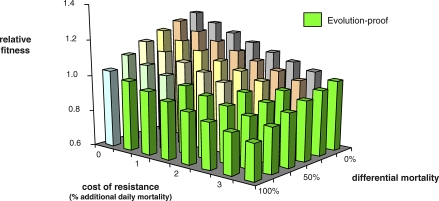
Fitness of Resistant Mosquitoes Relative to Susceptibles for an LLA Insecticide for Various Costs of Resistance and Differential Efficacy against Malaria-Infected Mosquitoes When relative fitness is greater than 1, resistance spreads, when relative fitness is less than 1, resistance can never spread, even when present in a population (complete evolution-proofing). Plotted values are for an LLA insecticide which kills mosquitoes on contact during or after their fourth gonotrophic cycle; these remove 94.2% of infectious mosquitoes when first deployed. Differential mortality is the proportionate reduction in mortality for uninfected mosquitoes compared to malaria-infected mosquitoes. Costs of resistance accrue as additional daily mortality rates. Relative fitness for conventional insecticides is 6.5 (Figure 1), which is little affected by costs resistance (see text). For model details and parameter values, see Materials and Methods.

The cost of resistance required to get evolution proofing is lowered for LLA insecticides which are disproportionately effective against malaria-infected mosquitoes (Figure 3). For instance, a four-cycle LLA insecticide, which is half as likely to kill uninfected mosquitoes, requires a cost of resistance of just 2.3% to be completely evolution proof. Strikingly, if its effectiveness against uninfected mosquitoes was just 10% of what it was against infected mosquitoes, complete evolution proofing would occur at a resistance cost of just 0.9%, a cost which would be barely measurable. An LLA insecticide that kills only malaria-infected mosquitoes is completely evolution proof for vanishingly small costs of resistance (0.43%).

We are aware of only one quantitative estimate of the relative fitness of resistant mosquitoes in the field. This comes from the non-malarial vector Culex pipiens, following 40 years of organophosphorous (OP) insecticide spraying in Southern France [48,63]. There, the fitness of individuals homozygous for a resistance mutation relative to sensitive homozygotes is 0.63–0.72 (discussed further in Text S1). Using our model to calculate lifetime fecundity of mosquitoes experiencing various mortality costs of resistance in the absence of treatment, we find that the relative fitness associated with the highest cost of resistance required to get complete evolution proofing, 3.4% additional mortality, is 0.78; the corresponding figures for the 2.3% and 0.9% additional mortality described above are 0.84 and 0.93, respectively. Similar figures are obtained if we assume the costs of resistance accrue as reduced fecundity rather than reduced adult survival (unpublished data). Thus, the costs of resistance required to achieve complete evolution proofing are not out of line with those seen in nature.

## Product Options

The foregoing analysis argues that new insecticides for malaria control should minimize impact on mosquito lifetime reproductive output while also minimizing the number of infectious mosquitoes. The achievement of this goal ideally requires insecticides that kill late in life, that are disproportionately effective against malaria-infected mosquitoes, and for which resistance carries fitness costs. This approach, which will retard the spread of resistance alleles (possibly forever) even when they are already present in a population, should complement or even replace strategies aimed solely at delaying the initial origin of resistance, since these latter strategies often have no effect when resistance eventually becomes established in a population.

We are unaware of any attempts to evaluate potential insecticides for these properties, but it is possible to imagine a range of approaches or modes of action that would achieve late-life killing. For example, cumulative exposure to ordinarily sublethal doses of an insecticide over multiple feeding cycles could result in the death of older mosquitoes. Alternatively, formulation techniques such as microencapsulation could provide a means for slow release of an insecticide over time. Similarly, age-dependent mortality could be achieved by exploiting the fact that in Anopheles, metabolic detoxification activity declines with age [29,64]. This decline may be a natural consequence of senescence and explain why Anopheles become more susceptible to DDT, malathion, and pyrethroids with increasing age [64–68]. It is also easy to imagine compounds that would act disproportionately on mosquitoes with advanced malaria infections. Malaria parasites impose large metabolic costs on mosquitoes [69–73], either directly via competition for resources, or indirectly by prompting costly immune responses. These costs are likely to increase as the malaria infection progresses, both as a consequence of the increasing parasite burdens as replication proceeds, and as blood and other meals become progressively less successful as the mouthparts become blocked with sporozoites [74]. Metabolically stressed insects should be more vulnerable to normally sublethal doses or compounds.

An even more radical possibility is that there may be formulations or deployment strategies that would convert existing insecticides into evolution-proof LLA insecticides. As noted above, DDT, pyrethroids, and malathion are disproportionately efficacious against mosquitoes that are old enough to transmit malaria [64–68]. Doses lower than those currently recommended may therefore be insufficient to kill younger mosquitoes but fatal to older, near-infectious mosquitoes. If so, existing insecticides could be evolution-proofed by changing concentrations delivered in the field, even where resistance is currently spreading in a population.

The evolutionary benefits of an LLA insecticide apply irrespective of the resistance mechanism involved, but the greatest benefits accrue for compounds against which resistance is the most costly. Resistance to conventional insecticides involves target site alterations, metabolic detoxification, and behavioral avoidance [2,12,13]. It seems highly likely that the fitness costs of resistance will depend on the mechanisms involved. In other insects, there is evidence that fitness costs depend on the insecticide, and for some but importantly not all, the costs can clearly be negligible or degrade through time as modifiers spread [63,75]. Explicit deployment of compounds against which resistance is costly would be a novel approach and would also assist traditional resistance management strategies.

There may also be ways of achieving evolution-proof insecticides by means other than chemicals. For example, fungal biopesticides are already known to generate the required phenotypes. These insecticides are based on oil-formulated spores of entomopathogenic fungi applied to surfaces on which adult mosquitoes will rest after blood feeding [46,76,77]. Although still at a research stage, they have proven to be very effective malaria transmission blockers in the laboratory [76] and can be delivered in African houses [77]. Fungal biopesticides work as time-dependent late-life insecticides, killing the insect 7–14 d post-contact [46,76–79]. They are also disproportionately effective against malaria-infected mosquitoes [76]. Other biocontrol agents such as Wolbachia [80] and densoviruses [81] have a similar potential to disproportionately target older mosquitoes [82], and hence are potentially immune to the evolution of host resistance.

Moreover, nothing in our arguments actually requires compounds that kill mosquitoes. Critical is that older, infectious mosquitoes be prevented from biting humans. Killing them is one way of doing this, but analogous arguments would apply to products which, late in life, have other transmission-blocking effects, such as interference with host-seeking behavior, flight, or blood feeding propensity. Sublethal effects like these must have pronounced fitness consequences for mosquitoes but, as with lethality, these need not result in strong selection for resistance so long as they impact in later life. Fungal biopesticides reduce feeding propensity as infection progresses [76,83]. Irritancy is an important feature of the protection offered by some existing chemical insecticides like pyrethroids, because it drives mosquitoes out of houses and in search of other hosts [33]. For highly anthrophilic species, like An. gambiae, evolution-proofing an irritant would require that it be selectively excito-repellent to older mosquitoes. For vector species that are not particularly anthrophilic, an insecticide that achieved irritancy without lethality would impose negligible selection for resistance at any age if the fecundity and survival of mosquitoes feeding on nonhuman hosts was no lower.

## Complications and Possible Downsides

Exploiting the ideas advocated above requires that criteria used to evaluate insecticides for malaria control be broadened beyond those currently now in use. Current minimum target product profiles required by the WHO Pesticide Evaluation Scheme for Phase 1 (laboratory) testing of insecticides for ITN and IRS use are 80% mortality up to 24 h post-exposure in young (2–5 d post-emergence) adult female Anopheles [84,85]. These thresholds, little changed since the 1960s [86], are used by the WHO to determine which insecticides to recommend to national authorities, and consequently by others to determine candidate compounds for inclusion in product development portfolios (for example, the Innovative Vector Control Consortium; http://www.ivcc.com/workwithus/application_process/irs.htm; accessed 4 March 2009). However, these “young-kill” criteria will result in the use of insecticides that impose near maximal selection for resistance. Minimizing that selection while still providing malaria control requires the use of insecticides and application protocols that impose marked reductions in transmission potential while simultaneously minimizing reductions in mosquito fitness. Assessing that requires exposing cohorts of young and old mosquitoes to insecticides, and analyzing life-long life tables, propensity to blood feed and, critically, fecundity, all ideally done with malaria-infected mosquitoes going through regular gonotropic cycles.

**Box 1. A Contemporary Example of the Selection of Insecticide Resistance by Indoor Residual Spraying**

Some of the best data on the impact of malaria control insecticides on resistance in Anopheles come from the Pacific Coast of Chiapas, Mexico [25,92,93]. In this region, agricultural use of insecticides around mosquito breeding sites together with indoor residual spraying of DDT for malaria control resulted in high levels of resistance to organochlorines, organophosphates, carbamates, and pyrethroids by the end of the 1970s. In the 1980s and ‘90s, DDT continued to be used for malaria control, and DDT resistance remained at high levels. However, the agricultural use of insecticides declined markedly, so that by the mid-1990s, resistance to all other classes of insecticides had regressed to the point where it was barely detectable in standard WHO bioassays [93]. Genetic and biochemical analyses confirmed that, nonetheless, several known resistance alleles remained in these populations.

In the latter half of the 1990s, a 24-village IRS trial was conducted, aimed at evaluating the effect of contrasting resistance management strategies on the evolution of resistance [25,92,93]. This trial was prompted by rising concerns that the practice of using insecticides until resistance became a limiting factor was rapidly eroding the number of insecticides available for malaria control. Villages were assigned to one of four treatments of repeated cycles of house-spraying: (i) two spray applications per year of DDT, or three applications per year of (ii) a pyrethroid, (iii) a spatial mosaic of an organophosphate and a pyrethroid, or (iv) an annual rotation of an organophosphate, a pyrethroid, and a carbamate.

**Figure 4:**
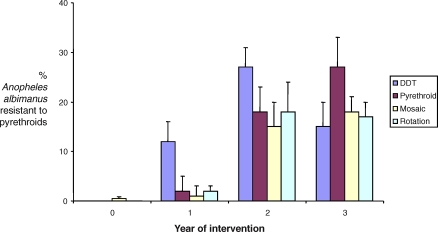
Evolution of Insecticide Resistance over Three Years of Indoor Residual Spraying in Coastal Southern Mexico. Pyrethroid resistance was assessed using WHO susceptibility bioassays before intervention (year 0) and over three years of different insecticide spraying regimes. Redrawn from [25].

Over the three years of the trial, pyrethroid resistance increased markedly in the mosquito populations in all villages, irrespective of insecticide treatment (Figure 4). Thus, spray campaigns targeting mosquitoes in an age-independent manner can very rapidly drive resistance evolution when relevant alleles are present in a population. Presumably, the majority of mosquitoes in all villages would have been resistant had the trial continued a few more years. This trial was well resourced and monitored, so that the insecticide coverage achieved was likely to be as high is practically possible, and thus representative of an IRS campaign confering maximal possible malaria control.

Resistance measures based on forcefully exposing mosquitoes to insecticide, such the WHO bioassays used to generate the data in the figure, likely under estimate epidemiologically relevant resistance because they can not assay important forms of resistance such as behavioral avoidance. Moreover, even resistance to direct exposure can be due to many different mechanisms and there can be many genetic variants in any one biochemical pathway. Thus, the contribution of any particular allele to overall resistance varies substantially. In this trial, levels of cytochrome P^450^, a major determinant of resistance to pyrethroids, were maintained at high levels only in villages sprayed solely with pyrethroids. In villages sprayed with DDT or subject to the rotation scheme, cytochrome P^450^ levels declined below detectability [25]. This suggests that cytochrome P^450^-mediated resistance can be managed by switching to a different insecticide class, but also that such switches need not limit resistance at the whole-insect level (Figure 4). It is our contention that evolution-proofing is possible for all resistance mechanisms, even where they already exist in a population, by targeting older mosquitoes.

Such experiments are not technically demanding, but they are logistically challenging, so that it would be impractical to do such tests for thousands of candidate compounds. However, for a limited number of promising candidates, such tests are feasible [46,76–79]. Candidates could be chosen in a number of ways. First, highly lethal compounds already at an advanced stage of development (or even registered) could be tested at lower concentrations for LLA properties. Second, known compounds, possibly rejected in previous screens because of slow speed of kill, could be revisited. Third, other product evaluation criteria such as likely cost, environmental safety, and potential for cross resistance could be used to preselect candidates for LLA testing from among the thousands of compounds currently tested in standard protocols. With lower lethality as a requirement, many more compounds might become feasible public health tools. We note that when it costs >US$175 million to bring a new compound into use [10], even substantially higher initial development costs for one LLA product look good against the costs of having to develop a second and third conventional insecticide (potentially ad infinitum if malaria can not be eradicated or controlled some other way). They also look good against the indefinite implementation costs and logistic constraints of resistance management strategies such as rotations or mosaics, which are currently being investigated as a means to prolong the life of existing, fast-acting insecticides once resistance is present (Box 1).

One side effect of the highly lethal insecticides currently in use for malaria control is that they also kill nontargets, such as nuisance mosquitoes and bedbugs. This side-effect is believed to help with householder compliance and uptake [3,37,87], at least before the nontargets also evolve resistance. LLA insecticides would not have these immediately beneficial side effects (although a product with differential impact against primary targets and secondary targets is a possibility). As such, LLA insecticides would essentially be community-level interventions, like transmission-blocking vaccines, with the associated issues of user take-up. Accordingly, it maybe that LLA insecticides will require delivery mechanisms that provide some degree of personal protection against nuisance insects, like bed nets, or imaginative, culturally-sensitive delivery systems and education programs that facilitate adoption irrespective of immediate personal relief from biting insects.

The late-life killing insecticides we are proposing here work because of the time Plasmodium takes to develop in mosquitoes. Could these insecticides select more rapidly developing parasites [82,88]? They might, but the short lives of mosquitoes must already be imposing intense natural selection for shorter extrinsic incubation periods, a selection pressure that must be further exacerbated by conventional insecticides. The apparent lack of response to this selection implies that significant fitness gains result from prolonged development [46,89], gains which presumably accrue through increased infectiousness [74]. It might be that LLA insecticides would add sufficient additional selection to offset these, but if it did, the resulting evolution would presumably generate substantially less-fit malaria parasites. Further investigation of this possibility is certainly warranted; in the meantime we note that the hypothetical evolution of significantly less-infectious parasites must be of less public health significance than the observed failure of existing insecticides in the face of resistance evolution.

For equivalent levels of coverage (at least lower than 100%), conventional insecticides will always give better control initially, before any resistance evolution. This disparity widens as coverage drops (unpublished data). Indeed, if only poor coverage can be achieved, the control benefits of LLA insecticides may be negligible. However, in that case, the need for them is also negligible, because resistance evolution is much less of a problem at low coverage, where insecticides of any type will impose weaker selection for resistance. LLA insecticides come into their own when coverage is high, an explicit aim of ITN and IRS programs, particularly in intense transmission areas. At high coverages, sustained reductions in transmission of ~95% by an LLA insecticide will quickly out weigh the even higher reductions that are initially possible with conventional insecticides once resistance against the latter inevitably spreads. Even LLA insecticides which fall short of being completely evolution-proof will minimize the evolutionary pressures that otherwise rapidly erode the efficacy of conventional insecticides. Very much slower rates of increase of resistance give more time for surveillance to detect resistance problems (or less frequent surveillance to provide the same warning), and more time to react. Lower selection pressures can also translate into many decades of additional effective control, which from a practical control perspective may be essentially infinite.

## Concluding Remarks

Somewhat ironically, given that all the insecticides currently in use in the public health sector derive from products developed for the agricultural sector, the long-term sustainability of LLA insecticides could be further enhanced precisely because they are likely to have little utility in agriculture. The linkage between public health and agricultural use of insecticides plagues public health use of insecticides like DDT and pyrethroids, where agricultural applications are one of the major drivers of resistance in vector populations [13,17,90]. This linkage could be broken by choosing LLA insecticides which could not be profitably reformulated for agricultural use, and for which there is no cross-resistance with existing agricultural pesticides. Moreover, restricted to the much smaller public health arena, any environmental impact of LLA insecticides would also be substantially reduced. However, an insecticide exclusive to public health would be unable to exploit the financial drivers promoting investment in agricultural insecticides, and so would need an artificially constructed market of the sort necessary to encourage the pharmaceutical industry to invest in malaria vaccines.

Our argument that public health insecticides can be evolution-proofed will not generalize to all vector-borne diseases, but it may be applicable to others with extrinsic incubation periods that approach the life spans of their vectors. Such diseases may include dengue, filariasis, West Nile virus, Japanese encephalitis, onchocercaisis, and Chagas disease. Novel technologies directed against a variety of disease vectors, such as those exploiting genetic modification of mosquitoes and selfish genetic elements, could also be immune to the evolution of host resistance if they are late-life acting.

The Global Malaria Action Plan (GMAP) [10] has laudable ambitions of spraying 172 million houses annually, and distributing 730 million insecticide-impregnated bed nets by the year 2010. If implemented with existing insecticides, this program will impose unprecedented selection for resistance. The historical record [22], and theory (e.g., Figure 1) shows that the medium-term prognosis for the insecticides currently in use is inescapably poor. Transitioning to an LLA insecticide strategy could see the benefits of the massive GMAP effort sustained, and could maintain for the long term the contribution of several key vector control tools to the goal of eradication. Failure to address evolution now runs the risk of replaying history [22]: operational disaster and a derailing of the whole malaria control agenda.

## Materials and Methods

The aim is to compare the relative effects of various hypothetical insecticides on (i) malaria transmission and (ii) evolution of resistance. Age-structured models of vector-borne diseases are notoriously difficult to parameterize, but because our aim is comparison of insecticides (our aim is theoretical proof-of-principle), and not absolute rates or amounts, considerable simplification is possible.

Our analysis consists of two parts: a static deterministic feeding cycle model (FCM) similar to those used by others [42–44], and a population genetics model (PGM). The FCM tracks, for each gonotrophic cycle over the lifetime of a mosquito (up to a maximum of ten cycles), probabilities of survival, contact with insecticides, frequency and ages of malaria infections, and the number of eggs laid. Incorporation of relevant mortality assumptions allows the FCM to assess the impact of a particular insecticide on the average lifetime number of infectious bites per mosquito and the average fecundity per mosquito. The PGM then uses the survival, infectious bite, and fecundity figures from the FCM for each class of mosquito to calculate, for the population as a whole, the relative frequency of resistant mosquitoes (our measure of resistance evolution) and the average number of infectious bites per mosquito (our measure of control), over a series of time periods (each equivalent to the length of one gonotrophic cycle), using standard population genetics approaches.

The FCM makes the following assumptions.

1. Mosquitoes bite humans randomly and uniformly.
2. Malaria-infected mosquitoes never become uninfected.
3. The proportion of humans who are infectious is constant.
4. A variety of parameters do not change over successive gonotrophic cycles: (i) the background mosquito mortality rate (what Smith and McKenzie [44] call “force of mortality”), which is considered as a constant per-capita daily death rate (i.e. there is no senescence), (ii) the probability of taking a blood meal and (iii) the probability of feeding on a human.
5. Conventional insecticides are instant kill.

LLA insecticides are envisaged to kill in either of two ways: (i) when they contact a mosquito after she has been through a fixed number of gonotrophic cycles, e.g., a four-cycle age-dependent insecticide (ADI) kills mosquitoes that have been through four or more cycles; or (ii) a fixed number of cycles after first contact, as might be the case for an infectious agent, e.g., a four-cycle time-delay insecticide (TDI) kills mosquitoes four cycles after initial contact. We have modeled both; the values we report are for ADIs. In Table S1, we show that ADIs and TDIs have equivalent effects. [Note that a mode of action for an LLA insecticide could also be via bioaccumulation, where lethal concentrations of an insecticide are finally achieved after repeated contacts over course of a mosquito's life. We have not explicitly modeled that mode of action].

The non-mathematical description of the model, considering ADIs only, is as follows. Female mosquitoes are followed from successful emergence through ten gonotrophic cycles. In each cycle, the probabilities of survival are tracked through the processes of host seeking, feeding, resting, finding an oviposition site, and laying. For each cycle, the proportion of mosquitoes that acquire a malaria infection, bite whilst infectious for malaria, and successfully lay eggs is also recorded. The mosquito may die whilst searching for a host, with a probability arising from the time spent searching and the background mortality rate. If she survives searching, she then attempts to feed on a human with a given probability, and on a nonhuman with one minus that probability. She may die whilst attacking the host immediately before or immediately after feeding, with probabilities calculated from the underlying risk of death when attacking a host, and the probability of encountering an insecticide (conventional or ADI) that kills on contact. Of those that successfully feed on a human host, females carrying a mature malaria infection give an infectious bite, whilst those so far uninfected may become infected, with a fixed probability. Those that survive feeding may then die during resting with a probability calculated from the time spent resting, and the background mortality rate. Those surviving resting may die whilst searching for a resting site, again depending on time and relevant mortality rates, and survivors may then die whilst attempting to lay, either before or after laying, with fixed probabilities. The tracked values give the proportion of mosquitoes surviving, biting, and laying in each cycle.

The variables and parameters used in the FCM to generate the figures reported in the paper are given in Table S2 with equations in Protocol S1. Differential mortality of malaria-infected and uninfected mosquitoes was calculated by applying only a proportion of the mortality associated with a given treatment to individuals not infected with malaria. The full mortality is applied to malaria-infected individuals. The model was implemented in Microsoft Excel [91].

The PGM makes the following assumptions:

1. Adult mosquito population size is constant.
2. Mosquitoes do not complete more than ten gonotrophic cycles.
3. The genetic make-up of mating males in any cycle is the same as that calculated for newly hatched mosquitoes in that cycle.
4. Males of all resistant/susceptibility genotypes are equally likely to mate successfully.
5. Females mate once only, in their first cycle, as is the norm [45].
6. Number of eggs produced per laying female is unaffected by egg paternal genotype.
7. Genotypes of emerging adults joining the population are in the same proportions as the genotypes of the generation of eggs from which they hatch.
8. Resistance is dominant, as can be the case [52].
9. Costs of resistance are dominant.
10. The proportion of infectious humans is constant.

Variables and parameter values for the PGM are given in Table S3 and associated equations are given in Protocol S2. The model uses survival probabilities from the FCM to calculate the initial age structure within the susceptible phenotypes in the population. The resistant allele is assumed initially to be present in heterozygotes, forming a very small proportion of the population, as detailed in Table S3. Subsequent spread of the allele reflects the age-linked survival probabilities for susceptible mosquitoes in the presence of the treatment and for resistant individuals, as well as the age-linked fecundity of each, all calculated in the FCM. The model, implemented in Microsoft Excel [91], analyses the changing status of the population for 1,290 sequential discrete time periods, each equivalent to the length of one feeding cycle.

Further discussion of model assumptions and sensitivity analyses are given in Text S1, together with additional analysis of the merits of the approach.

## Supporting Information

Protocol S1 Mathematical Details of the Feeding Cycle Model (19 KB PDF).

Protocol S2 Mathematical Details of the Population Genetics Model (19 KB PDF).

Table S1 The Equivalence of Time-Dependent and Age-Dependent Insecticides (10 KB PDF).

Table S2 Variables and Parameters of the Feeding Cycle Model (24 KB PDF).

Table S3 Variables and Parameters of the Population Genetics Model (19 KB PDF).

Text S1 Additional Discussion of Assumptions (24 KB PDF).

## Abbreviations

ADI: age-dependent insecticide
DDT: dichloro-diphenyl-trichloroethane
FCM: feeding cycle model
ITN: insecticide treated nets
IRS: indoor residual spraying
IVCC: Innovative Vector Control Consortium
LLA: late-life–acting
PGM: population genetics model
TDI: time-delay insecticide
WHO: World Health Organization
