# Supplementary material for: How to Make Evolution-Proof Insecticides for Malaria Control
Source: PLoS Biol. 2009 Apr 7;7(4):e1000058. doi: 10.1371/journal.pbio.1000058 (PMC3279047; doi:10.1371/journal.pbio.1000058)
Supplement: Protocol S1 — (19 KB PDF). [file pbio.1000058.sd001.pdf]

Protocol S1. Mathematical details of the Feeding Cycle Model

Using the symbols in Table S2, the calculation of average mosquito survival probabilities, normalized egg production and average infectious bites per cycle is as follows.

$$F_i = \frac{\left(\sum_{m=0}^{i-1} f_{i,m} v_{i,m}\right)}{V_i}$$

$$S_i = \frac{\left(\sum_{m=0}^{i-1} s_{i,m} v_{i,m}\right)}{V_i}$$

$$u = \sum_{i=1}^{10} I_i V_i$$

$$I_i = 0 \qquad i \le D$$

$$I_i = \frac{\sum_{m=D}^{i-1} q_{i,m,2} v_{i,m} + q_{i,m,3} v_{i,m}}{V_i} \qquad i > D$$

$$f_{i,m} = L\left(\sum_{h=1}^{3} q_{i,m,h}\right) z_{i,m}$$

$$V_1 = 1$$

$$V_i = \sum_{m=0}^{i-1} v_{i,m} \qquad i>1$$

$$v_{1,0} = 1.00$$

$$v_{i,0} = v_{i-1,0}\left(q_{i-1,0,1} + q_{i-1,0,2} + q_{i-1,0,3}\left(1-M\right)\right) z_{i-1,0} \qquad i > 1$$

$$v_{i,1} = v_{i-1,0} q_{i-1,0,3} M z_{i-1,0} \qquad i > 1$$

$$v_{i,m} = v_{i-1,m-1}\left(q_{i-1,m-1,1} + q_{i-1,m-1,2} + q_{i-1,m-1,3}\right) z_{i-1,m-1}$$

$$i > 1 \quad m > 1$$

$$s_{i,m} = \left(\sum_{h=1}^{3} q_{i,m,h}\right) z_{i,m} \qquad i < 10$$

$$q_{i,m,1} = He^{-rb}\left(1-a_1\right)$$

$$q_{i,m,2} = (1-p)(1-H)e^{-rb}(1-a_1)(1-k_i\delta)$$

$$q_{i,m,3} = p(1-H)e^{-rb}(1-a_1)(1-k_i\delta)$$

$$z_{i,m} = (1-a_2)e^{-r(\phi+\eta)}$$
