# Supplementary material for: How to Make Evolution-Proof Insecticides for Malaria Control
Source: PLoS Biol. 2009 Apr 7;7(4):e1000058. doi: 10.1371/journal.pbio.1000058 (PMC3279047; doi:10.1371/journal.pbio.1000058)
Supplement: Protocol S2 — (19 KB PDF). [file pbio.1000058.sd002.pdf]

Protocol S2. Mathematical details of the Population Genetics Model
Using the symbols detailed in Table S3, the calculation of the spread of resistant phenotypes in the population was as follows.

$$R_n = G_{3,n} + G_{2,n} d$$

where

$$G_{g,n} = G_{g,n-1} P_{g,n-1} + (1 - L_{n-1}) N_{g,n} \quad n>0$$

$g = 1 \rightarrow j = 1$
$g = 2 \rightarrow j = 1 + d$
$g = 3 \rightarrow j = 2$

$$P_{g,n} = \sum_{i=1}^{9} C_{g,n,i} S_{j,i}$$

$$L_n = \sum_{g=1}^{3} P_{g,n} G_{g,n}$$

$$N_{g,n} = E_{g,n-l} \quad n > l$$

$$N_{g,n} = E_{g,1} \quad n \leq l$$

$$C_{g,n,1} = \frac{N_{g,n}(1-L_{n-1})}{N_{g,n}(1-L_{n-1}) + P_{g,n-1}} \qquad n > 0$$

$$C_{g,n,i} = \frac{C_{g,n-1,i-1}S_{j,i-1}}{N_{g,n}(1-L_{n-1}) + P_{g,n-1}} \qquad 1 < i \quad n > 0$$

$$E_{g,n} = \frac{B_{g,n}}{B_{1,n} + B_{2,n} + B_{3,n}}$$

$$B_{1,n} = \sum_{i=1}^{10} (F_{1,i}C_{1,n,i}G_{1,n} + 0.5F_{1+d,i}C_{2,n,i}G_{2,n})A_{1,n+1-i}$$

$$B_{2,n} = \sum_{i=1}^{10} F_{1,i}C_{1,n,i}G_{1,n}A_{2,n+1-i} + 0.5F_{1+d,i}C_{2,n,i}G_{2,n} + F_{2,i}C_{3,n,i}G_{3,n}A_{1,n+1-i}$$

$$B_{3,n} = \sum_{i=1}^{10} (F_{2,i}C_{3,n,i}G_{3,n} + 0.5F_{1+d,i}C_{2,n,i}G_{2,n})A_{2,n+1-i}$$

$$A_{1,n} = (0.5 f_2 N_{2,n} + f_1 N_{1,n}) / (f_1 N_{1,n} + f_2 N_{2,n} + f_3 N_{3,n})$$

$$A_{2,n} = (0.5 f_2 N_{2,n} + f_3 N_{3,n}) / (f_1 N_{1,n} + f_2 N_{2,n} + f_3 N_{3,n})$$

Efficacy of treatment is given as

$$T_n = 1 - M_n / u_s$$

where

$$M_n = \sum_{g=1}^{3} \sum_{i=1}^{10} C_{g,n,i} I_{j,n,i} G_{g,n} \quad .$$
