# Supplementary material for: How to Make Evolution-Proof Insecticides for Malaria Control
Source: PLoS Biol. 2009 Apr 7;7(4):e1000058. doi: 10.1371/journal.pbio.1000058 (PMC3279047; doi:10.1371/journal.pbio.1000058)
Supplement: Table S1 — (10 KB PDF). [file pbio.1000058.st001.pdf]

Table S1. Time-dependent insecticides and age-dependent insecticides have equivalent effects on disease transmission and resistance evolution. Comparisons are made at 80% exposure, with mortality occurring 4 cycles after contact (time-dependent inseciticide, TDI) or in mosquitoes aged 4 cycles or older (age-dependent insecticide, ADI).

	Cycle number									
	**C1**	**C2**	**C3**	**C4**	**C5**	**C6**	**C7**	**C8**	**C9**	**C10**
Time-dependent insecticide	**No mortality from treatment, no mosquitoes carrying TDI for 4 cycles**			Of the mosquitoes surviving to cycle 4, 80% were exposed to TDI in cycle 1, and will now die as a result	Of the mosquitoes surviving to cycle 5, 80% were exposed to TDI in cycle 2, and will now die as a result	**and so on......**				
Mortality in cycle from LLA	0.0%	0.0%	0.0%	80%	80%	80%	80%	80%	80%	80%
Age-dependent insecticide	**No mortality from treatment, no mosquitoes 4 cycles or older**			Of the mosquitoes surviving to cycle 4, 80% are now exposed to ALI, of which all are 4 cycles of age or older, and will die as a result	Of the mosquitoes surviving to cycle 5, 80% are now exposed to ALI, of which all are 4 cycles of age or older, and will die as a result	**and so on......**				
Mortality in cycle from LLA	0.0%	0.0%	0.0%	80%	80%	80%	80%	80%	80%	80%
