# Supplementary material for: How to Make Evolution-Proof Insecticides for Malaria Control
Source: PLoS Biol. 2009 Apr 7;7(4):e1000058. doi: 10.1371/journal.pbio.1000058 (PMC3279047; doi:10.1371/journal.pbio.1000058)
Supplement: Table S2 — (24 KB PDF). [file pbio.1000058.st002.pdf]

Table S2. Variables and parameters for the Feeding Cycle Model.

Variable or Parameter	Symbol	Value or Constraints	Source (where relevant)
Base instantaneous mortality rate per day	r	0.12	a
Length of gonotrophic cycle (days)	w	2.85	a
Time spent host searching and feeding during a cycle (days)	b	1.26	b
Time spent finding oviposition site and laying during a cycle (days)	ϕ	1.26	b
Length of resting period (days)	η	0.32	b
Time required for parasite sporogonic development (days)	d	10.78	a
Proportion human population infectious for malaria	p	0.04	a,c
Probability attacks non-human host	H	0.17	a
Probability killed when attacking host before biting	a_1	0.05	d
Probability killed when attacking host after biting (excluding mortality from insecticide treatments)	a_2	0.05	d
Probability becomes infected with malaria when biting infectious human host	M	0.80	
Cycle number *(identifies specific cycle in the ten cycles over which average probabilities are tracked in the FCM)*	i	$0 \le i \le 10$	
Probability contacts and is killed by instant action (conventional or age-dependent) treatment when attacking human host, before biting	k_i	*for conventional chem* $k_i = 0.80$ i=1,2..10 *for ALI* $k_i = 0$ i<effective age $k_i = 0.8$ i≥effective age	
Malaria status, the number of whole or partial cycles since infection with malaria	m	$0 \le m \le 10$ $m = 0$ *means not infected*	
Differential mortality factor	δ	$\delta = 1$ when $m>0$ $0 \le \delta \le 1$ when $m=0$	
Type of host attacked	h	h=1, non-human h=2, non-infectious human	

Variable or Parameter	Symbol	Value or Constraints	Source (where relevant)
		h=3, infectious human	
Normalised number of eggs laid per successfully laying mosquito per cycle	L	100	
Average normalised number of eggs laid in cycle *i* by mosquitoes surviving to the start of cycle *i*	F_i		
Average normalised number of eggs laid in cycle *i*, by mosquitoes starting cycle *i* with malaria status *m*	$f_{i,m}$	$m<i$	
Average probability of survival from start of cycle *i* to start of cycle *i*+1	S_i		
Average probability that a mosquito starting cycle *i* with malaria status *m*, will survive to start of cycle *i*+1	$s_{i,m}$	$m<i$	
Average probability of a mosquito being alive at start of period *i*.	V_i		
Average probability of a mosquito being alive, with malaria status *m* at start of period *i*.	$v_{i,m,}$	$m<i$	
Probability that a mosquito alive at start of cycle *i* with malaria status *m*, survives and bites host type *h* in cycle *i*	$q_{i,m,h}$	$m<i$	
Probability that a mosquito alive at start of cycle *i* with malaria status *m* having survived to bite, then survives to lay eggs	$z_{i,m,}$	$m<i$	
Average number of infectious bites in cycle *i* per mosquito alive at the start of cycle *i*	I_i		
Average lifetime number of infectious bites per mosquito	u		
Time, measured in whole units equal to length of sporogonic cycle, from infection of mosquito to cycle from which mosquito gives infectious bites	D	$0<D\leq10$	

a. Average value, based on data from four foci of intense malaria [1]
b. Assuming *c.*11.1% of every cycle is spent resting (8 hours in a 72 hour cycle), with the rest of the gonotrophic cycle divided equally between laying and feeding
c. Derived from overall probability biting human host will result in malaria infection in mosquito [1]
d. Based on 0.10 mortality during attack [2], assuming equal probabilities of death before and after a feed.

References

1. Killeen GF, McKenzie FE, Foy BD, Schieffelin C, Billingsley PF, et al. (2000) A simplified model for predicting malaria entomologic inoculation rates based on entomologic and parasitologic parameters relevant to control. *American Journal of Tropical Medicine and Hygiene* 62: 535-544.

2. Killeen GF, Smith TA (2007) Exploring the contributions of bed nets, cattle, insecticides, and excitorepellency to malaria control: a deterministic model of mosquito host-seeking behaviour and mortality. *Transactions of the Royal Society of Tropical Medicine and Hygiene* 101: 867-880.
