# Supplementary material for: How to Make Evolution-Proof Insecticides for Malaria Control
Source: PLoS Biol. 2009 Apr 7;7(4):e1000058. doi: 10.1371/journal.pbio.1000058 (PMC3279047; doi:10.1371/journal.pbio.1000058)
Supplement: Table S3 — (19 KB PDF). [file pbio.1000058.st003.pdf]

Table S3. Variables and parameters for Population Genetics Model. Time periods are equal in length to gonotrophic cycles, but we use cycles to refer to units of mosquito age and periods to refer to units of time.

Variable or Parameter	Symbol	Value or Constraints
Period number (periods over which the population is tracked)	n	$0 \leq n \leq 1290$
Mosquito age (gonotrophic cycles)	i	$0 < i \leq 10$
Phenotype	j	susceptible $j = 1$<br>resistant $j = 2$
Probability of survival for mosquitoes with phenotype j, to age i+1 from age i (i>=1)	$S_{j,i}$	*values from FCM*
Number of periods between egg laying and adult emergence	l	**3**
Dominance of resistance allele	d	dominant $d = 1$<br>recessive $d = 0$
Genotype (normal allele s, resistant allele r)	g	(s,s) g = 1<br>(s,r) $g = 2$<br>(r,r) $g = 3$
Allele a as proportion alleles contributed by male population in period n	$A_{a,n}$	s $a = 1$<br>r $a = 2$
Proportion of mosquitoes with genotype g which survives from period n to period n+1	$P_{g,n}$	
Proportion of mosquitoes with genotype g which are age i at start of period n	$C_{g,n,i}$	
Normalised average number of eggs laid by females of phenotype j, aged i	$F_{j,i}$	*values from FCM*
Total normalised number of eggs with genotype g laid in period n	$B_{g,n}$	
Proportion of all eggs laid in period n having genotype g	$E_{g,n}$	
Proportion of all new adults having genotype g at start of period n	$N_{g,n}$	$\boldsymbol{N_{2,1}=10^{-9}}$
Proportion of total population having genotype g at start of period n	$G_{g,n}$	$\boldsymbol{G_{1,0} = 1-G_{2,0}}$<br>$\boldsymbol{G_{2,0} = 10^{-9}}$<br>$\boldsymbol{G_{3,0} = 0}$
Proportion of the population surviving period n	L_n	
Proportion of the population resistant at start of period n	R_n	
Fitness factor for males with genotype g	f_g	**1.00**
Average normalised number of infectious bites per mosquito of phenotype j aged i in period n	$I_{j,n,i}$	*values from FCM*
Average normalised number of infectious bites per mosquito per cycle in a susceptible population, in absence of treatment	u_s	*value from FCM*
Average normalised number of infectious bites per mosquito in population in period n	M_n	
Efficacy of treatment in period n	T_n	
