# Supplementary material for: How to Make Evolution-Proof Insecticides for Malaria Control
Source: PLoS Biol. 2009 Apr 7;7(4):e1000058. doi: 10.1371/journal.pbio.1000058 (PMC3279047; doi:10.1371/journal.pbio.1000058)
Supplement: Text S1 — (24 KB PDF). [file pbio.1000058.sd003.pdf]

Text S1. Additional discussion of assumptions

The model framework we have used here is designed to allow comparisons of the control and evolutionary outcomes of insecticides with different modes of action: relative performance is assessable, but the model is inadequate for predicting absolute time lines or impact on human morbidity and mortality. One key model assumption is that the human malaria rate (proportion of people infectious with malaria) is constant. We note that the effect of this assumption is to underestimate the relative public health benefits of LLA insecticides. Conventional insecticides have little room for improvement (in the scenarios modeled in Figs 1-3, they reduce infectious mosquitoes by 99.8% from the outset), whereas initial control benefits of LLA insecticides can improve as malaria rates fall in the human population. Such changes, and the problems of knowing what alternative strategies will be implemented once conventional insecticides fail, is also why we have not attempted to compare insecticides using some measure of cumulative transmission over the lifetime of a given product. Another assumption is that total mosquito densities are unaffected by the insecticides. Conventional insecticides do clearly reduce mosquito densities [e.g. 1] but, again, this can have little impact on the near perfect control they exert before resistance begins to evolve. LLA insecticides would be unlikely to significantly reduce overall mosquito numbers.

Any model of vector-borne diseases is parameter and assumption rich. We performed sensitivity analyses on the following to assess the significance of various assumptions. In all cases, key conclusions were unchanged by alterations in the given parameters within biologically sensible ranges, although in some cases a 3-cycle killer optimized the combination of malaria control and evolution-proofing.

- Prevalence of malaria in the human population
- Coverage (% exposure to insecticide treatments)
- Combined effects of coverage and prevalence of malaria in the human population.
- Separate analyses for each of the four different geographical sites [2] which we averaged to get the parameter values used in the model outputs reported in the paper
- Genetic make-up of males in each cycle matching that of female population or of new adults only
- Costs of resistance accrue solely as reduced fecundity
- Recessivity of resistance and of costs of resistance. Clearly evolution proceeds more slowly if resistance is recessive, but because comparison of different insecticides is the key output, our conclusions are qualitatively the same if we assume recessivity

We also made a number of other assumptions that bear comment.

We assumed that insecticides do not affect vector densities. It seems likely that LLA insecticides acting on older age classes only may indeed have negligible impact on vector population sizes, since they will eliminate only the fecundity of older mosquitoes, and those mosquitoes, being relatively rare, will contribute negligibly to mosquito population growth rates. In contrast, conventional insecticides are used to suppress *Anopheles* densities so that part of their effectiveness comes about by alterations in the vector:human ratio. Our conclusions regarding the relative initial control efficacy of conventional and LLA insecticides are nonetheless robust to violation of our assumption of constant mosquito densities because in the scenario we modeled, conventional insecticides provided a level of initial control that was so high it could only be very slightly improved by reductions in vector densities.

Our model assumes no mosquito senescence and no fitness effects of malaria infection. Yet mosquitoes do senesce [3-6] and malaria has pronounced effects on mosquito fitness, perhaps by reducing vector survival [7] but particularly by reducing host fecundity [8,9]. We note that both senescence and malaria-induced fitness

reductions will further enhance the evolution-proofing of insecticides which disproportionately kill old and/or malaria-infected mosquitoes. This is because any reductions in mosquito fitness through other factors reduce the relative fitness impact of insecticides, thus reducing selection for resistance. Alternatively, it could be that longer lived mosquitoes live longer because they have higher viability, and consequently more late-life reproduction. If this resulted in a higher proportion of their offspring produced later in life, this would strengthen selection for resistance in that fraction of the population transmitting malaria, perhaps slightly strengthening selection for resistance. We are currently investigating the effects of different assumptions about age-specific mortality and reproduction and a thorough analysis of these will be published elsewhere.

Complete evolution-proofing can be achieved if there are high enough costs of resistance. The actual magnitude of the costs of insecticide resistance in *Anopheles* are unclear; there has been remarkably little work done on the topic considering the critical role costs of resistance play in conventional resistance management. The quantitative estimate we give in the main text is the only estimate of the relative fitness of resistant mosquitoes in the field of which we are aware. This comes from the non-malarial vector, *Culex pipiens*, following 40 years of organophosphorous (OP) insecticide spraying in the Montepellier region of Southern France [10,11]. OP insecticides kill by inhibiting acetlycholinesterase in the central nervous system. As in *Anopheles* [12], resistance to OPs in *Culex* is encoded by a single amino acid mutation at position 119 of the *ace-1* locus. This mutation results in a 60% reduction in enzymantic activity, which probably underlies the variety of developmental and behavioural problems experienced by *Culex* mosquitoes with this mutation [10,11]. The frequency of the *ace-1*^{R} mutation declines across a transect running from an OP-treated region into an untreated region. The cost of resistance we discuss in the main text is the cost which Labbe et al. [10] estimate is required to account for the rate of decline in the frequency of the *ace-1*^{R} mutation across that transect. Costs of resistance can be eroded by the spread of compensatory mutations. There is little doubt that resistance evolution is continuing around the Montpellier region of Southern France, with new resistance alleles continuing to appear [10]. This means that the cost estimates we cite in the main text need not be the minimum evolution eventually achieves. Nonetheless, we note that the estimate we are using is that seen after 40 years of spraying, suggesting that costs might have been even higher once, and that simple compensatory mutations of large effect rendering resistance effectively costless do not appear readily.

Finally, a the slower evolution of resistance driven by LLA insecticides (Fig 1 in main paper) is not a consequence of weaker selection accruing from poorer initial control. For instance, a conventional insecticide at a coverage of 50.1% achieves an initial control of 94.2%, which is the same as that for the 4-cycle age-specific killer at 80% coverage reported in the paper. But even at that lower coverage, the conventional insecticide has a useful lifespan about 1/5 that of LLA at the higher coverage.

References.

1. Sharp BL, Ridl FC, Govender D, Kuklinski J, Kleinschmidt I (2007) Malaria vector control by indoor residual insecticide spraying on the tropical island of Bioko, Equatorial Guinea. Malaria Journal 6.
2. Killeen GF, McKenzie FE, Foy BD, Schieffelin C, Billingsley PF, et al. (2000) A simplified model for predicting malaria entomologic inoculation rates based on entomologic and parasitologic parameters relevant to control. American Journal of Tropical Medicine and Hygiene 62: 535-544.
3. Clements AN, Paterson GD (1981) The analysis of mortality and survival rates in wild populations of mosquitoes. Journal of Applied Ecology 18: 373-399.
4. Harrington LC, Buonaccorsi JP, Edman JD, Costero A, Kittayapong P, et al. (2001) Analysis of survival of young and old *Aedes aegypti* (Diptera : Culicidae) from Puerto Rico and Thailand. Journal of Medical Entomology 38: 537-547.

5. Okech BA, Gouagna LC, Killeen GF, Knols BGJ, Kabiru EW, et al. (2003) Influence of sugar availability and indoor microclimate on survival of *Anopheles gambiae* (Diptera : Culicidae) under semifield conditions in western Kenya. Journal of Medical Entomology 40: 657-663.
6. Styer LM, Carey JR, Wang JL, Scott TW (2007) Mosquitoes do senesce: Departure from the paradigm of constant mortality. American Journal of Tropical Medicine and Hygiene 76: 111-117.
7. Ferguson HM, Read A (2002) Why is the impact of malaria parasites on mosquito survival still unresolved? Trends in Parasitology 18: 256-261.
8. Hurd H (2001) Host fecundity reduction: a strategy for damage limitation? Trends in Parasitology 17: 363-368.
9. Ahmed AM, Hurd H (2006) Immune stimulation and malaria infection impose reproductive costs in *Anopheles gambiae* via follicular apoptosis. Microbes and Infection 8: 308-315.
10. Labbé P, Berticat C, Berthomieu A, Unal S, Bernard C, et al. (2007) Forty years of erratic insecticide resistance evolution in the mosquito *Culex pipiens*. PLoS Genetics 3: 2190-2199.
11. Raymond M, Berticat C, Weill M, Pasteur N, Chevillon C (2001) Insecticide resistance in the mosquito *Culex pipiens*: what have we learned about adaptation? Genetica 112: 287-296.
12. Weill M, Lutfalla G, Mogensen K, Chandre F, Berthomieu A, et al. (2003) Insecticide resistance in mosquito vectors. Nature 423: 136-137.
